# Katy Did and Katy Didn’t

**Published:** 1874-04

**Authors:** 


Katy Did, and Katy Didn't.—*An Inquiry concerning Priority in the Ligation of the Internal Carotid Artery.* By William K. Bowling, M.D., Emeritus Professor of the Theory and Practice of Medicine, in the Medical Department of the University of Nashville. Nashville, Tennessee: 1874. Pamphlet, pp. 20.

